# Care burden among Iranian family caregivers of patients with schizophrenia: the predictive role of quality of life and life satisfaction

**DOI:** 10.3389/fpsyt.2025.1559786

**Published:** 2025-05-12

**Authors:** Maede Esmaeeli, Seyedmohammad Mirhosseini, Somaye Minaei-Moghadam, Saeed Ghasempour, Mohammad Hasan Basirinezhad, Hossein Ebrahimi

**Affiliations:** ^1^ Student Research Committee, School of Nursing and Midwifery, Shahroud University of Medical Sciences, Shahroud, Iran; ^2^ Department of Nursing, School of Nursing and Midwifery, Shahroud University of Medical Sciences, Shahroud, Iran; ^3^ School of Nursing and Midwifery, Guilan University of Medical Sciences, Rasht, Iran; ^4^ Nursing and Midwifery Care Research Center, Mashhad University of Medical Sciences, Mashhad, Iran; ^5^ Department of Epidemiology and Biostatistics, School of Public Health, Shahid Sadoughi University of Medical Sciences, Yazd, Iran; ^6^ Center for Health Related Social and Behavioral Sciences Research, Shahroud University of Medical Sciences, Shahroud, Iran

**Keywords:** caregiver burden, quality of life, life satisfaction, schizophrenia, caregiver

## Abstract

**Introduction:**

Caring for patients with schizophrenia poses significant challenges for families. This study examined the relationships between caregiving burden, quality of life, and life satisfaction among family caregivers.

**Methods:**

This cross-sectional study was conducted in 2023–24 in Mashhad, Iran. Family caregivers who provided care to a patient with schizophrenia for at least six months completed the Zarit Burden Inventory to evaluate caregiver burden, the 12-item Short Form Health Survey to assess quality of life, and the Satisfaction with Life Scale to measure life satisfaction. The data were analyzed using multiple linear regression.

**Results:**

A total of 211 family caregivers participated, with a mean age of 48.17 ± 14.98 years, of whom 130 (61.61%) were female. Caregiver burden was associated with caregiver-related factors, such as lower life satisfaction (β = -1.27, p < 0.001, 95% CI = -1.48, -1.06), employment status, where housewives experienced lower caregiving burden than unemployed individuals (p = 0.039, β = -4.4, 95% CI = -8.65, -0.21), and marital status, where singles experienced lower caregiving burden than married individuals (p = 0.001, β = -7.89, 95% CI = -11.88, -3.90). In addition, patient-related factors such as longer duration of illness (p < 0.001, β = 0.42, 95% CI = 0.20, 0.64) and lack of health insurance coverage (compared to having coverage) (p = 0.023, β = 5.10, 95% CI = 0.71, 9.49) were associated with higher caregiver burden. Together, these variables explained 62.9% of the variance in the total care burden score.

**Conclusion:**

The findings of this study showed that the majority of family caregivers of patients with schizophrenia experienced moderate to severe levels of caregiving burden. This burden was associated with lower levels of life satisfaction, employment status, and marital status of the caregiver, as well as longer duration of illness and lack of health insurance coverage for the patient.

## 1 Introduction

Schizophrenia presents as a challenging psychiatric condition affecting roughly one percent of the global population (1, 2). This disorder is multifaceted, displaying positive symptoms like hallucinations and delusions, alongside negative symptoms such as blunted affect, apathy, and impaired social functioning (3–5). Owing to factors like the lack of acceptance, self-care, insight, and judgment among many patients with schizophrenia, family caregivers assume primary responsibility for their care (6, 7). Statistics indicate that around 50% to 70% of patients with schizophrenia receive ongoing care from their families (8). Family caregivers provide both financial and psychological assistance, dedicating significant time to their loved one’s care while grappling with the perpetual fear of disease recurrence and its repercussions on other family members (9). However, these long-term caregiving duties drain family members’ energy, fostering negative emotions like hopelessness, guilt, depression, and helplessness (10). Additionally, caregivers face immense pressure due to the severity of their loved one’s symptoms, leading to emotional distress and encountering unpredictable stressors such as erratic behavior, impulsivity, and disruptions in daily life, compounded by societal stigma and discrimination (11). Consequently, caregivers bear a substantial burden (12, 13).

Caregiving burden encompasses the physical, emotional, social, and economic challenges faced by family members caring for their loved ones (14–17). A literature review by Hajebi et al. (18) revealed that over 70% of Iranian family caregivers of patients with schizophrenia experienced above-average levels of caregiving burden (18). Similarly, Rahmani et al. (19) found that nearly 87% of these caregivers reported moderate to severe levels of burden in Iran (19). These findings underscore the pervasive nature of caregiving burden among family caregivers of individuals with schizophrenia, highlighting the need for further exploration of associated factors. Meanwhile, the burden of caregiving is greatly influenced by cultural aspects of societies. Cross-cultural research has shown that caregiving practices vary between Western and Eastern countries (20). For instance, in Asian countries where formal facilities and social services are limited, family caregivers rely heavily on family resources and support (21). Additionally, in Iranian society, unique cultural teachings, religious beliefs, and strong family ties contribute to significant caregiving by family members (22). This underscores the importance of evaluating caregiving burden and identifying related factors in family caregivers of individuals with schizophrenia in Iranian society, considering its specific cultural context.

Previous studies have highlighted the impact of quality of life on the caregiving burden experienced by family caregivers (7, 23–26). The World Health Organization (WHO) defines quality of life as an individual’s perception of their position in life, framed by the culture and value systems in which they live, and in relation to their goals, expectations, standards, and concerns (27). Caring for a family member with severe psychiatric disorders often compromises caregivers’ quality of life, potentially leading to negative consequences for their caregiving abilities and, ultimately, the quality of care provided to the patient (28). Moreover, caregivers’ diminished quality of life may impede the family’s ability to make informed decisions regarding treatment plans and emotional support, indirectly impacting the well-being of patients with psychiatric disorders, including schizophrenia (7, 28, 29). Consequently, it is vital to address the quality of life of caregivers, as it can serve as a predictor of overall life satisfaction (30).

Life satisfaction emerges as another factor intertwined with the caregiving burden within the context of schizophrenia (31), closely linked to quality of life (30). This construct reflects individuals’ overall assessment of various aspects of their lives, such as work, relationships, health, and leisure activities (32). Furthermore, life satisfaction serves as a crucial metric to monitor, given its predictive value for future physical and mental health outcomes (30, 33). As previously noted, the responsibility of caring for individuals with schizophrenia imposes a significant burden on family caregivers, potentially leading to social isolation and financial strain, contributing to psychological distress (34, 35). These challenges may precipitate mental health disorders and influence caregivers’ overall life satisfaction (36, 37). Hence, the provision of care appears to exert adverse effects on caregivers’ life satisfaction (38). However, limited research has explored this relationship, particularly concerning family caregivers of patients with schizophrenia.

Previous studies have shown a two-way relationship between caregiving burden, quality of life, and life satisfaction, with each variable exerting a mutual influence (26, 39, 40). However, limited research has specifically examined the complex interplay among caregiving burden, quality of life, and life satisfaction in this caregiver population. This gap underscores the need for updated and comprehensive insights in this area. The present study adds novelty by investigating caregiving burden as a dependent variable, with quality of life and life satisfaction as independent variables. This research aims to explore the associations between caregiving burden, quality of life, and life satisfaction among family caregivers of individuals with schizophrenia.

## 2 Materials and methods

### 2.1 Study design and settings

A total of 211 family caregivers of individuals with schizophrenia participated in this cross-sectional study, conducted from December 2023 to March 2024. Family caregivers were recruited from those recently discharged from Ebne-Sina Psychiatric Hospital in Mashhad, Iran. Ebne-Sina Psychiatric Hospital, located in Khorasan Razavi Province, is the largest referral center for psychiatric care in eastern Iran. Eligible participants were enrolled using a convenience sampling method.

### 2.2 Participants

At the final stage of the discharge process, patients and their caregivers were referred to the hospital’s family education unit. Here, the researcher assessed their eligibility by reviewing inclusion and exclusion criteria and conducting interviews. If conditions were favorable, family caregivers were then included in the study. All family caregivers in this study were required to meet specific inclusion criteria: their patients had to have a confirmed diagnosis of schizophrenia by a psychiatrist (as documented in the medical record), the patient must have experienced symptoms for at least six months (41), the caregiver had to be at least 18 years old, and the caregiver must have provided care for a minimum of six months (42). Exclusion criteria included caregivers with diagnosed psychiatric disorders that significantly impair daily functioning (e.g., schizophrenia, bipolar disorder, or major depressive disorder with psychotic features) (43), those with diagnosed substance use disorders (44), or those using neuroleptic drugs, based on self-reported information. Additionally, four caregivers were excluded from the analysis because their caregiving duration was less than six months.

### 2.3 Data collection

After securing the necessary approvals, the study objectives were initially communicated to all in this study, data collection tools comprised demographic information questionnaires, Diener’s Life Satisfaction Scale (SWLS), SF-12 Quality of Life Questionnaire, and Zarit Burden Inventory (ZBI), all administered as self-report measures that they were utilized widely to assess the care burden, quality of life and life satisfaction among family caregivers of patients with schizophrenia. The selected tools for measuring the study’s main variables (quality of life, caregiving burden, and life satisfaction) have demonstrated acceptable validity and reliability in previous research (45–47). Participants were instructed to complete the questionnaires in a quiet environment, away from distractions, provided they were literate.

#### 2.3.1 Demographic information form

The demographic information form comprises two sections: one pertaining to caregiver details (including their relationship with the patient, daily hours of caregiving, and presence of familial history of mental illness), and the other focusing on patient information (such as health insurance coverage, history of substance abuse, alcohol addiction, aggression, suicide and homicide attempts, psychotic symptoms, and illness duration). Additionally, variables including age, marital status, employment status, education level, gender, receipt of secondary support, and underlying medical conditions were assessed for both patients and caregivers. In this study, employment status was classified into six categories: unemployed, employed, housewife, self-employed, retired, and student, following the commonly recognized framework in Middle Eastern societies (42, 48). Self-employment encompasses occupations such as shopkeeping, construction, and carpentry, where individuals utilize their financial resources or specialized skills to offer goods and services independently. Conversely, employment refers to individuals working within structured organizations, administrative systems, or companies (either on a part-time or full-time basis) while receiving a fixed salary. Furthermore, the term “unemployed” denotes individuals actively seeking work but not currently holding a job, whereas “housewife” refers to those primarily responsible for managing household affairs, including childcare, cooking, and domestic upkeep. However, in Middle Eastern societies, not all married women are classified as housewives, as some also participate in paid employment alongside their domestic responsibilities (49). Psychotic symptoms were extracted from definitive information noted in the medical records and the patients’ individual records in their electronic health records.

#### 2.3.2 Zarit Burden Inventory

In this study, the Zarit Burden Inventory was utilized to assess caregivers’ burden of care. This inventory consists of 22 items, each scored on a five-point Likert scale (ranging from “Never” = 0 points to “Always” = 4 points). To derive the total score, the scores of all questions are summed, yielding a range of 0 to 88 points. Scores falling below 30 indicate mild burden, scores between 31 and 60 signify moderate burden, and scores between 61 and 88 denote severe burden. Consequently, higher scores correspond to greater caregiver burden. Zarit et al. (50) reported reliability coefficients for this inventory using the test-retest method and internal consistency (Cronbach’s alpha) as 0.71 and 0.91, respectively (50). The Persian version of the ZBI demonstrated good reliability, with reporting an internal consistency of 0.90 using Cronbach’s alpha coefficient (16). The internal consistency of the ZBI in this study was confirmed with a Cronbach’s alpha coefficient of 0.731.

#### 2.3.3 12-item Short Form Health Survey

In this study, the SF-12 quality of life questionnaire developed by Ware et al. (51) was employed to assess participants’ quality of life. This 12-item questionnaire evaluates two overarching subscales: physical health and mental health. The physical health subscale comprises items pertaining to physical functioning, physical role, bodily pain, general health, social functioning, vitality, role limitations due to emotional problems, and perceived mental health. The SF-12 comprises two dimensions: Physical quality of life and Mental quality of life. Physical quality of life is measured by items 1, 2, 3, 4, 5, and 8, while Mental quality of life is measured by items 6, 7, 9, 10, 11, and 12. Each dimension has a possible score range of 6 to 24. So, scores on this questionnaire range from 12 to 48, with higher scores indicating better quality of life. Scores falling between 37 and 48 indicate good quality of life, scores between 25 and 36 signify moderate quality of life, and scores between 12 and 24 denote poor quality of life. Reliability analysis conducted by Ware and colleagues (51) using the test-retest method. Cronbach’s alpha coefficients ranging from 0.89 to 0.76, indicating acceptable reliability levels for this questionnaire (51). Additionally, Montazeri et al. (52) assessed the validity and reliability of the Persian version of this questionnaire. Exploratory factor analysis revealed two main factors: physical and psychological. Confirmatory factor analysis confirmed the goodness of fit of the model. Internal consistency, assessed using Cronbach’s alpha coefficient, yielded coefficients of 0.73 for the physical factor and 0.72 for the mental factor (52). The SF-12 demonstrated reliable internal consistency in this study, with a Cronbach’s alpha coefficient of 0.745.

#### 2.3.4 Satisfaction with Life Scale

In this study, participants’ life satisfaction was assessed using the Satisfaction with Life Scale (SWLS) developed by Diener et al. This scale comprises 5 items designed to gauge individuals’ overall appraisal of life. Responses to the items are scored on a seven-point Likert scale, ranging from “completely disagree” (scored as 1) to “strongly agree” (scored as 7). The total score is obtained by summing the scores of all items, resulting in a minimum score of 5 and a maximum score of 35. Higher scores indicate greater life satisfaction. Diener and colleagues (53) reported a Cronbach’s alpha coefficient of 0.87 for their scale, indicating good internal consistency (53). The Persian version of this scale has demonstrated acceptable validity and reliability in Iranian society, as evidenced by previous studies (54, 55). In this study, the SWLS exhibited reliable internal consistency, with a Cronbach’s alpha coefficient of 0.787.

### 2.4 Sample size

The sample size was determined based on standard deviations reported in previous studies that examined key variables, including quality of life, caregiving burden, and life satisfaction (56–58). Using these parameters, we initially calculated a sample size of 195 participants to achieve 80% statistical power at a 0.05 significance level. To account for potential attrition, we applied a 10% adjustment, bringing the final target sample size to 215. The calculation followed the standard formula:


$$n=\frac{z^{2}*(p*1-p)}{d^{2}}$$

where z represents the critical value for a 95% confidence level (1.96), p is the estimated proportion based on previous research, and d is the margin of error. Despite these adjustments, the final study sample comprised 211 participants due to unforeseen exclusions and participant dropout. However, this slight reduction was unlikely to affect the study’s statistical power or the reliability of its findings.

### 2.5 Data analysis

The collected data were analyzed using descriptive statistical measures, including frequency, percentage, mean, and standard deviation. Inferential statistical tests, specifically multiple linear regression analysis (backward method), were conducted using SPSS software. Initially, univariate linear regression models were applied individually for all variables under investigation. Subsequently, variables demonstrating significance levels below 0.2 were selected for inclusion in the multiple model. To assess collinearity among the studied variables, the Variance Inflation Factor (VIF) was calculated using STATA software, with values below 3 indicating the absence of collinearity. A significance level of less than 0.05 was considered for all statistical tests.

### 2.6 Ethical considerations

The research adhered to the principles outlined in the Declaration of Helsinki, ensuring participants’ rights to voluntary participation, non-harm, withdrawal from the study, and confidentiality of information. Prior to participation, both verbal and written informed consent were obtained from all participants. Additionally, the researchers are committed to upholding the principles set forth by the Committee on Publication Ethics (COPE) when disseminating the study findings. The study received approval from the Ethics Council of Shahroud University of Medical Sciences under the code IR.SHMU.REC.1401.143.

## 3 Results

A total of 211 family caregivers of patients with schizophrenia were assessed. The average caregiver age was 48.17 years (SD = 14.98). The majority were women (61.6%), and 20% (n = 40) were single. Regarding their relationship with the patient, 25% (n = 53) were siblings. Caregivers reported providing an average of 7.19 hours of care per day (SD = 4.16). A detailed summary of caregiver demographics is provided in **Table 1**.

**Table 1:** Demographic characteristics of caregivers of patients with schizophrenia.

Variable		N	%
Sex	Male	81	38.39
	Female	130	61.61
Marital status	Married	171	81.04
	Single	40	18.96
Educational level	Secondary school	103	48.82
	High School	51	24.17
	Academic degree	57	27.02
Underlying disease	No	117	55.45
	Yes	94	44.55
Job status	Unemployed	13	6.16
	Housewife	83	39.34
	Self-employed	67	31.75
	Retired	27	12.80
	Employed	16	7.58
	Student	5	2.37
Coverage by supportive organizations	Yes	33	15.64
	No	178	84.36
Relation with patient	Parent	24	11.38
	Children	99	46.92
	Wife/husband	35	16.59
	Sibling	53	24.32
Family history of psychiatric disorder	Yes	41	19.43
	No	170	80.57
		Mean	SD
Age (years)		48.17	14.98
Daily caregiving hours		7.19	4.16

%, percent; SD, Standard deviation; N, frequency.

The patients had an average age of 39.01 years (SD = 9.99), with an average of 11.16 years (SD = 6.98) since initial psychiatric diagnosis. The most frequently reported psychotic symptoms were hallucinations (67.77%) and delusions (82.94%). Additionally, recent aggression was documented in 183 patients, and 35.07% (n = 74) had a record of a recent suicide attempt. Further clinical details are summarized in **Table 2**.

**Table 2:** Demographic profile of individuals diagnosed with schizophrenia.

Variable		N	%
Sex	Male	122	57.82
	Female	89	42.18
Marital status	Married	143	67.77
	Single	68	32.23
Educational level	Secondary school	120	56.87
	High School	74	35.07
	Academic degree	17	8.06
Underlying disease	No	182	97.33
	Yes	5	2.67
Job status	Unemployed	140	66.35
	Housewife	33	15.64
	Self-employed	30	14.22
	Retired	4	1.90
	Employed	1	0.47
	Student	3	1.42
Health insurance coverage	Yes	181	85.78
	No	30	14.22
Family history of psychiatric disorder	Yes	41	19.43
	No	170	80.57
Hallucination	Yes	143	67.77
	No	68	32.23
Delusion	Yes	175	82.94
	No	36	17.06
Disorganized behavior	Yes	155	73.46
	No	56	26.54
Disorganized speech	Yes	161	76.30
	No	50	23.70
Negative symptoms	Yes	177	83.89
	No	34	16.11
History of substance abuse	Yes	67	31.75
	No	144	68.25
History of alcohol consumption	Yes	6	2.84
	No	205	97.16
		Mean	SD
Age (years)		48.17	14.98
Duration of illness (year)		7.19	4.16

%, percent; SD, Standard deviation; N, frequency.

Approximately 48.82% of participants reported experiencing moderate quality of life, while 22.75% described their caregiving burden as severe. For clarification, moderate quality of life was defined as a score between 25 and 36, and severe caregiving burden was characterized by a score ranging from 61 to 88. Additional findings regarding the main variables are presented in **Table 3**.

**Table 3:** Mean scores of quality of life, burden and life satisfaction among caregivers of patients with schizophrenia.

Variables		N	%
Care burden	Low	39	18.48
	Moderate	124	58.77
	Severe	48	22.75
Quality of life	Poor	16	7.58
	Average	103	48.82
	Good	92	43.60
		Mean	Standard deviation
Quality of life		34.12	5.05
Physical		15.45	14.38
Mental		18.67	1.90
Life satisfaction		17.82	8.00
Caregiver burden		45.97	17.48

n, Frequency; %, Percent.

In the examination of variables influencing caregiver burden, a univariate linear regression model was initially used to assess the relationship of all variables. Variables with a significance level below 0.2 were included in the multivariate model. The results showed an R value of 0.79, R² of 62.9%, an F value of 33.97, and a p-value < 0.001. Using a backward elimination approach, 62.9% of the variance in caregiver burden was explained by the included variables. The findings of this study indicated that the Variance Inflation Factor (VIF) for the entire regression model was 1.12, suggesting no collinearity among the variables. Single caregivers reported a significantly lower burden than married caregivers (β = -7.89, 95% CI: -11.88 to -3.90, p < 0.001). Housewives experienced a significantly lower burden than unemployed caregivers (β = -4.43, 95% CI: -8.65 to -0.21, p = 0.039). Caregivers whose patients had health insurance reported a significantly lower burden (β = -5.10, 95% CI: 0.71 to 9.49, p = 0.023). Each additional year of disease duration was associated with an increase in caregiver burden (β = 0.42, 95% CI: 0.20 to 0.64, p < 0.001). Additionally, for every one-unit increase in life satisfaction score, the caregiver burden score significantly decreased (β = -1.27, 95% CI: -1.48 to -1.06, p < 0.001). Further details are provided in **Table 4**.

**Table 4:** Examining the impact of predictive variables on the burden of care through multiple regression analysis.

Variables		β	SE	t	(95% CI)	p
Intercept		57.92	3.16	18.29	(51.68, 64.17)	<0.001
Marital status	Married	Reference				
	Single	-7.89	2.02	-3.90	(-11.88, -3.90)	<0.001
Daily caregiving hours		1.07	0.20	5.15	(0.66, 1.47)	<0.001
Job status	Unemployed	Reference				
	Housewife	-4.43	2.14	-2.07	(-8.65, -0.21)	0.039
	Self-employed	-0.49	2.28	-0.21	(-4.99, 4.01)	0.830
	Retired	-4.88	5.60	-0.87	(-15.93, 6.17)	0.385
	Employed	4.02	11.11	0.36	(-17.88, 25.94)	0.717
	Student	-5.08	6.55	-0.78	(-18.00, 7.83)	0.438
Health insurance coverage	Yes	Reference				
	No	5.10	2.22	2.29	(0.71, 9.49)	0.023
Illness duration (per year)		0.42	0.11	3.75	(0.20, 0.64)	<0.001
Life satisfaction		-1.27	0.10	-11.90	(-1.48, -1.06)	<0.001

SE, Standard error; p, P value; CI, Confidence interval.

## 4 Discussion

Caring for individuals with severe and chronic mental illnesses, particularly schizophrenia, presents a prolonged journey fraught with numerous challenges for family caregivers. In such circumstances, caregivers may encounter unique conditions that significantly impact their overall well-being. Recognizing this, the present study was undertaken to explore the interplay between caregiving burden, quality of life, and life satisfaction among family caregivers of patients with schizophrenia.

The findings of the present study indicate that a significant number of caregivers experienced a moderate level of caregiving burden. These findings align with results from prior studies on assessing the caregiving burden for patients with schizophrenia (45, 59, 60). Although different assessment tools were utilized across these studies, they collectively underscore the escalating challenges faced by caregivers in the realm of severe psychiatric disorders, particularly schizophrenia.

The findings of this study indicate that about half of the caregivers reported a moderate quality of life, consistent with results from previous research (10, 59). The concept of quality of life is intricately linked to chronic pathological conditions requiring long-term management. Schizophrenia shares similarities with other chronic medical illnesses in terms of its impact on quality of life. However, distinctions exist, including the influence of psychopathology, symptoms, and medication side effects specific to schizophrenia, which can significantly diminish the quality of life of caregivers (61).

The results of the present study indicated that moderate levels of life satisfaction among family caregivers of patients with schizophrenia. Existing literature on life satisfaction among caregivers of individuals with psychiatric disorders is relatively limited. However, it is important to recognize that living with a person who has a mental illness can exert a detrimental impact on the physical and mental well-being of family members. When caregivers are required to provide continuous supervision or direct care, their life satisfaction may be significantly compromised (62).

The findings of this study suggest that higher burden of care was linked to a reduced level of life satisfaction among caregivers of patients with schizophrenia. Similarly, findings from Mazlan et al.’s (63) study in Malaysia indicated that caregivers with lower life satisfaction and lower monthly income, caring for patients with spinal cord injuries and associated cognitive and neurobehavioral disorders, were at a heightened risk of experiencing caregiving burden (63). These findings are consistent with observations in other domains. Other factors related to the life satisfaction of family caregivers of individuals with schizophrenia should be explored in a study by Rajendran et al. (64). The findings of this study revealed a strong positive correlation between self-esteem, support from others, and resilience, as well as a moderate positive correlation between self-efficacy, family support, and friend support with life satisfaction in Indian family caregivers of individuals with schizophrenia. Conversely, stress, anxiety, depression, and stigma showed a significant negative correlation with life satisfaction in Indian family caregivers of these patients (64). The findings of previous studies in the field of non-psychiatric disorders were also consistent with the results of the present study (65–67). Although no evidence of a bidirectional relationship between these variables was found, Fauziana et al. (56) considered positive aspects of caregiving (PAC) as a mechanism that links life satisfaction and caregiving burden in Singaporean caregivers of older adults. This suggests that an increase in life satisfaction following an increase in PAC can reduce caregiving burden (56). Based on the previous systematic review study, adopting appropriate strategies and approaches such as emotion-based activities, didactic emotional development, health promotion, social media, music, and multi-component interventions seems essential to promote life satisfaction (68). This can lead to both direct and indirect reductions in the burden of care by improving PAC (56).

As previously discussed, quality of life hinges on individuals’ life satisfaction, an aspect presumed to influence caregivers’ experiences significantly. However, contrary to expectations, the current study did not unveil a statistically significant relationship between quality of life and caregiving burden (23, 45, 59, 69–71). This contrasts with findings from extensive research indicating a notable inverse relationship between these two variables among caregivers of psychiatric patients. Despite the recognized importance of caregiver quality of life in schizophrenia patient care outcomes, it’s crucial to acknowledge that the absence of a significant relationship in the present study may stem from various factors. These factors could include a limited sample size or the timing of data collection immediately after discharge from an inpatient unit. The hospitalization process can offer a break for caregivers. Also the utilization of non-specific measurement tools for assessing caregiving burden and quality of life within the context of schizophrenia. The use of non-specific tools to measure quality of life in some societies exposes the results of the assessment to measurement error. Therefore, it should be noted that the results of the present study are subject to measurement bias (72). The absence of correlation in our study implies a lack of statistically significant relationship, but it does not negate the possibility of such a relationship existing in reality. This suggests that the current study design may not adequately capture the nuances of the relationship between these variables. Hence, future research endeavors are encouraged to address these limitations by employing larger sample sizes, allowing adequate time for data collection, or alternative study designs to explore the relationship between quality of life and caregiving burden in the realm of schizophrenia more comprehensively.

Among the variables associated with a higher burden of care among participants, the extended duration of the patient’s illness stands out. This observation aligns with previous research indicating a direct relationship between a longer illness duration and increased caregiving burden among caregivers of individuals with chronic psychiatric disorders, including schizophrenia, major depression, and bipolar disorder (73–75). Findings from studies in other domains, such as Parkinson’s disease, further corroborate the current results (76).

According to the findings of the current study, another patient-related variable associated with the burdensome caregiving experience of family caregivers is the hours of daily patient care. A direct and significant relationship was observed between longer hours spent providing care and a higher burden of care among caregivers. This observation is consistent with a previous study conducted in Pakistan, which also found a direct relationship between caregiving hours and the caregiving experience of caregivers of individuals with serious mental illnesses (73). It’s important to consider the impact of caregiving responsibilities on caregivers’ employment participation. Various factors, including the hours of care and the patient’s dependence on the caregiver, can influence caregivers’ ability to engage in work activities (77). Consequently, in the realm of psychiatric disorders, it can be anticipated that caregivers facing extended caregiving hours may experience heightened care burden (78).

The study findings also revealed that caregivers of patients with health insurance coverage experienced significantly less care burden compared to those without insurance. Lack of access to health insurance poses a significant barrier to accessing specialized mental health services (79). In the context of schizophrenia, the financial implications of caregiving encompass treatment expenses for the care recipient, providing financial assistance to the patient, and the caregiver’s loss of productivity and income (80). Prior research has indicated that patients’ access to health insurance coverage serves as a predictor of reduced care burden among family caregivers, aligning with the current study’s findings (81, 82).

The study also identified certain caregiver-related variables that correlated significantly with caregiving burden. Married caregivers, as revealed by the study, experienced a significantly higher burden of caregiving compared to their single counterparts. This finding is consistent with previous research, such as the study conducted by Siddiqui et al. (73), which similarly observed that married caregivers tended to experience greater burden (73). This trend has also been observed among family caregivers of individuals with bipolar disorder (75). Married caregivers typically juggle multiple roles and responsibilities in their personal lives, which could lead to conflicts in fulfilling their caregiving duties for a sick family member.

Furthermore, the caregiver’s employment status emerged as a significant factor influencing their caregiving experience. Specifically, unemployed individuals reported a substantially higher burden of care compared to caregivers who were housewives. This finding aligns with prior research demonstrating that the care burden among unemployed caregivers is notably elevated (83–85). One possible explanation for this observation is that housewives typically adhere to a structured daily routine involving tasks such as childcare, household chores, and shopping. This established routine provides them with a sense of control and self-management. As a result, they may possess a stronger sense of role identity compared to unemployed individuals who lack such structured duties and responsibilities.

The present study, while shedding light on the caregiving burden among family caregivers of patients with schizophrenia, is not without limitations. Its cross-sectional design and limited sample size warrant caution in interpreting the findings. To address these limitations, future research could employ longitudinal designs and include larger cohorts of caregivers. Moreover, this study was conducted in Iran, where cultural and social norms play a significant role in shaping caregiving experiences. In Iranian society, caregiving is often regarded as a moral and customary obligation, deeply rooted in Islamic teachings (86). These cultural expectations often compel caregivers to prioritize their loved ones’ needs over their own well-being. Additionally, the stigma associated with mental illness can intensify caregiver burden, emotional stress, and social isolation. These unique cultural factors make the findings of this study context-specific, limiting their generalizability to other settings. Additionally, reliance on self-report questionnaires leaves the results vulnerable to response bias. Furthermore, the study’s scope was limited to a select set of variables, omitting other potentially relevant factors such as resilience, coping strategies, and hope. Despite these constraints, this study contributes valuable insights into the challenges faced by family caregivers of patients with schizophrenia. Its findings lay the groundwork for future research endeavors, offering avenues for further exploration and understanding in this critical area of study.

### 4.1 Clinical implications

The present study underscores the necessity of implementing culturally tailored psychoeducational interventions that address the unique challenges encountered by Iranian family caregivers, including managing caregiving burden, enhancing coping skills, and alleviating emotional distress. Mental health support systems should integrate comprehensive programs focusing on stress management, self-care practices, problem-solving skills, and resilience-building strategies. Furthermore, the establishment of supportive policies and community-based resources is essential to alleviate caregiver burden and promote their overall well-being.

## 5 Conclusion

Caregivers of individuals with schizophrenia experience substantial caregiving burden, often associated with low levels of life satisfaction. As such, it is advisable to implement supportive measures aimed at enhancing caregivers’ quality of life and satisfaction. This can be achieved through psychoeducation interventions and bolstering social support provided by a multidisciplinary team. Additionally, integrating this form of support into schizophrenia care programs tailored specifically for caregivers is essential.

## Data Availability

The original contributions presented in the study are included in the article/Supplementary Material. Further inquiries can be directed to the corresponding author.
